# Onboard Carbon Capture for Circular Marine Fuels

**DOI:** 10.1021/acssuschemeng.4c08354

**Published:** 2025-02-04

**Authors:** Margarita A. Charalambous, Valentina Negri, Valentin Kamm, Gonzalo Guillén-Gosálbez

**Affiliations:** Institute for Chemical and Bioengineering, Department of Chemistry and Applied Biosciences, ETH Zurich, Vladimir-Prelog-Weg 1, 8093 Zurich, Switzerland

**Keywords:** onboard carbon capture, heavy duty transport, maritime emissions, container ship, synthetic fuels

## Abstract

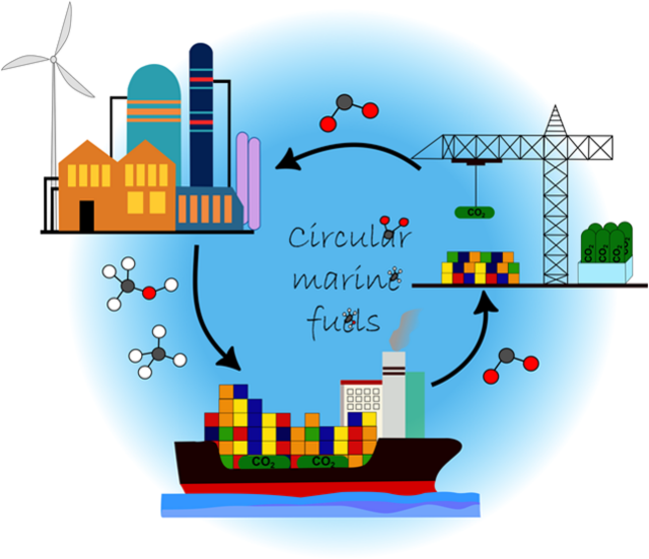

The transition to low- and zero-carbon fuels is the primary driver for reducing emissions in the maritime industry, with methanol and natural gas emerging as the most promising options. However, carbon-based fuels will continue to emit considerable amounts of pollutants during their use phase. This work explores the application of circular economy principles in the shipping industry by integrating carbon capture and utilization technologies. Specifically, we propose a closed-loop system where carbon dioxide (CO_2_) generated onboard is captured using a chemical absorption technology and stored until the ship reaches a port. The captured CO_2_ is then unloaded and transported to a fuel production facility, where it reacts with electrolytic hydrogen to regenerate the required propulsion fuel. Here, we evaluate the economic, technical, and future environmental viability of methanol and natural gas as circular marine fuels. Our findings indicate that natural gas could achieve a 65% reduction in CO_2_ emissions by 2050, while methanol could lead to 55%, both with a 91% carbon capture rate. However, there is an economic premium of 339 USD tonCO_2_^–1^ for methanol and 260 USD tonCO_2_^–1^ for natural gas. Additionally, the shift to circular fuels is expected to increase costs by a factor of 3–6 compared to conventional operations.

## Abstract 2

We evaluate the economic, technical, and future environmental viability of methanol and natural gas as circular marine fuels through carbon capture onboard.

## Introduction

Shipping is the backbone for global freight trade, and is projected to account for 10% in greenhouse gas (GHG) emissions by 2050 due to the reliance on fossil fuels like heavy fuel oil (HFO).^1^ The sectors’ current 3% share of global emissions, perceived as less critical compared to other sectors, and operations occurring beyond national boarders have delayed the regulatory action. The International Maritime Organization (IMO) only set GHG reduction targets in 2018, with stricter goals introduced in 2023 i.e., a 30% reduction by 2030 and carbon neutrality by 2050.^2,3^

The identified primary driver for cutting emissions is to shift to low and zero-carbon fuels, tailored to the specific needs of each shipping subsector.^4,5^ Ammonia, hydrogen (H_2_), and electricity are among the zero-carbon fuels that hold promise to halt tailpipe emissions, in particular the carbon emissions.^6,7^ Electrification is favored for its high engine efficiency and low costs, but is limited to small ships on shorter routes due to battery size and weight. H_2_, while technically feasible as shown in the report by the International Council on Clean Transportation on liquid H_2_ in internal combustion engines,^8^ faces challenges concerning high liquefaction costs, propulsion system costs (700 Euro kW^–1^),^9^ and storage space requirements, making it less attractive for deep sea shipping.^10,11^ Even though it holds significant environmental promise, as well as lower costs in the future, the safety risks related to hydrogen leakages (that are considered as GHG emissions),^12^ and the uncertain timeline for the development of H_2_ engines render it unsuitable for deployment in the short to midterm. Ammonia, instead, seems promising for large ships, i.e., above 150,000 dead weight tonnage (equivalent to 15,000 TEU),^13^ but requires extensive safety and emission reduction measures, with the first ammonia engine expected in 2025.^4,14,15^ For long-distance travel, low-carbon fuels like natural gas and methanol can offer a 10–25% reduction in carbonaceous combustion emissions, while more is known on these fuels related to bunkering and safety.^4^ Additionally, biofuels and synthetic fuels (such as electrofuels — e-fuels —) can provide further emission reductions depending on the fuel production pathway.^4,16^

Another emerging alternative is capturing CO_2_ in the emissions produced from carbon-based fuels and utilize or store it in underground reservoirs. Since its proposal in 2013, onboard carbon capture has gained attention,^17^ with the IMO considering it as a viable option, especially when integrated with carbon capture and utilization (CCU) and storage (CCS) value chains.^18,19^ Research has explored the technical and economic aspects of capturing CO_2_ from HFO and natural gas ships, but comprehensive environmental assessments of such framework are still lacking.^20−22^ Notably, post-combustion carbon capture using absorbents, which has a high technology readiness level (TRL), is currently one of the most suitable methods for ships, as it can effectively utilize engine waste heat for amine regeneration. Other technologies such as membrane separation, cryogenic separation,^23^ and calcium looping exist,^24^ yet they pose some challenges (i.e., high operational temperatures), making chemical absorption with amines particularly appealing at present.

Several works have looked at the life cycle impacts of marine applications, focusing on fossil fuels and biofuels.^25−27^ Moreover, carbon capture onboard HFO ships was assessed environmentally by Negri et al. applying the planetary boundaries framework, finding that capture onboard could make it easier for the shipping industry to operate within the safe operating space.^28^

Most of the said environmental studies typically use fixed background data taken from current environmental databases (reflecting the current upstream supply chains in the world economy). That is, they assume that the economic activities providing inputs to the main system (foreground system) will remain the same, thereby overlooking potential emission reductions linked to future decarbonization efforts and learning curves. Prospective life cycle assessment (pLCA) builds on the standard LCA to evaluate future environmental impacts, incorporating results of integrated assessment models (IAMs) to modify the background system.^29,30^ The *premise* python-base tool, developed by Sacchi et al., systematically modifies the background system to reflect the results of IAMs.^31^ To our best knowledge, Watanabe et al. is one of the two works that investigate the future environmental performance of biofuels for the shipping industry, showing 54% reductions in GHG emissions by 2050, with biomethane performing best.^32^ Ingwersen et al. used pLCA to compare the environmental impacts of electrofuels finding which ones can satisfy the FuelEU Maritime Regulation by 2050.^33^

While the general concept of CCU has been discussed in a broad sense, focusing mostly on CO_2_ from direct air capture (DAC) and point sources, we are unaware of any study on CCU to close the carbon loop within the shipping industry.^18^ In this work, we fill this gap by proposing a circular marine fuels concept that integrates carbon capture onboard ships fueled with CO_2_-based fuels, where the captured CO_2_ is used to produce the propulsion fuel. We carry out a technoeconomic and prospective environmental assessment of circular marine fuels and compare their performance with the business-as-usual scenario (BAU) of HFO ships. Our work identifies circular marine fuels as a viable option for reducing emissions in both the short- and long-term, while ensuring CO_2_ availability and lowering costs compared to a CCU alternative reliant fully on CO_2_ from DAC.

## Methodology

### Objective and Scope of This Study

**Figure 1:**
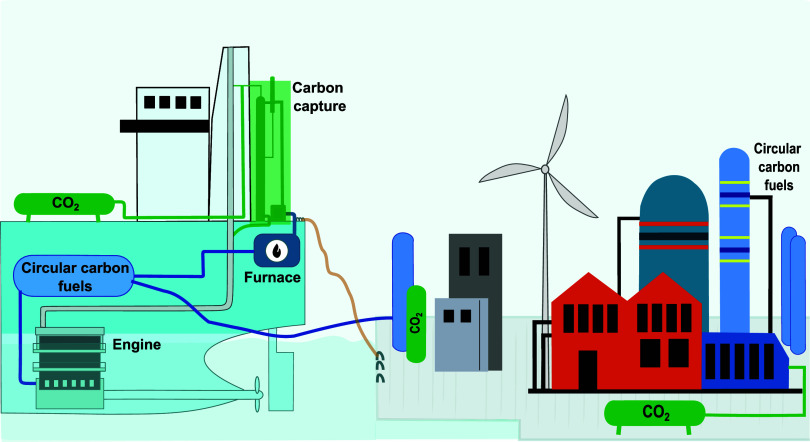
Set-up of study. The study objective is to apply circular economy principles in the shipping industry to reduce the carbon dioxide (CO_2_) emissions. We use carbon capture onboard ships that are powered with CO_2_-based fuels. We temporarily store CO_2_ on the ship, and at the port, we unload it and send it to a fuel production facility, where we generate the propulsion fuel. In this work, we retrofit a carbon capture plant on a container ship and also model the fuel production.

The system analyzed in our study is depicted in Figure 1. We assume that the captured CO_2_ is unloaded at the next port and transported to a fuel production facility where it will react with H_2_ to produce the propulsion fuels, thereby closing the carbon cycle within the shipping industry. We evaluate the circular concept using two low-carbon CO_2_-based fuels expected to be used in the future, namely methanol and natural gas.^4^ For both circular marine fuels, the propellant is also used for steam generation to eliminate the need for additional energy carriers onboard.

Our study focuses on container ships with a capacity of 3500 twenty-foot equivalent units (i.e., containers). According to a recent DNV report, container ships are considered the most suitable vessels for integrating onboard carbon capture due to their frequent and fixed routes.^18^ We use the Stena Germanica, a methanol-powered passenger ferry with 23,000 kW engine, as our reference ship.^34^ For the natural gas-powered ship, we assume the same ship size and calculate the fuel consumption based on the low heating value and engine efficiency. The designed carbon capture unit is intended to capture CO_2_ emissions from the engine and the furnace (required onboard for providing heat to the system).

For the part of our work modeled on land — fuel production —, we use the HFO demand for the port of Hamburg in Germany,^35^ as a reference, converting this demand to the equivalent amount for methanol and natural gas based on their low heating values. The final demand accounts for the fuel required for both the engine and the steam generation, while also accounting for 1% methane slip in the natural gas ship. The annual demand for methanol is 5 million tonnes per year and for natural gas 1.9 million tonnes per year. Although, these production plant sizes exceed the capacity of existing plants, we believe that centralized fuel production plants can benefit from economies of scale resulting in lower unitary production costs, ultimately making the future maritime industry more cost-effective. Additionally, locating these plants close to ports can reduce transportation costs and emissions, therefore reducing the complexity of the supply chain and bringing the production close to the point of demand. Last, both fuels are produced from electrolytic H_2_ powered with wind electricity, while additional requirements for CO_2_ are satisfied using DAC.

### Process Modeling

**Figure 2:**
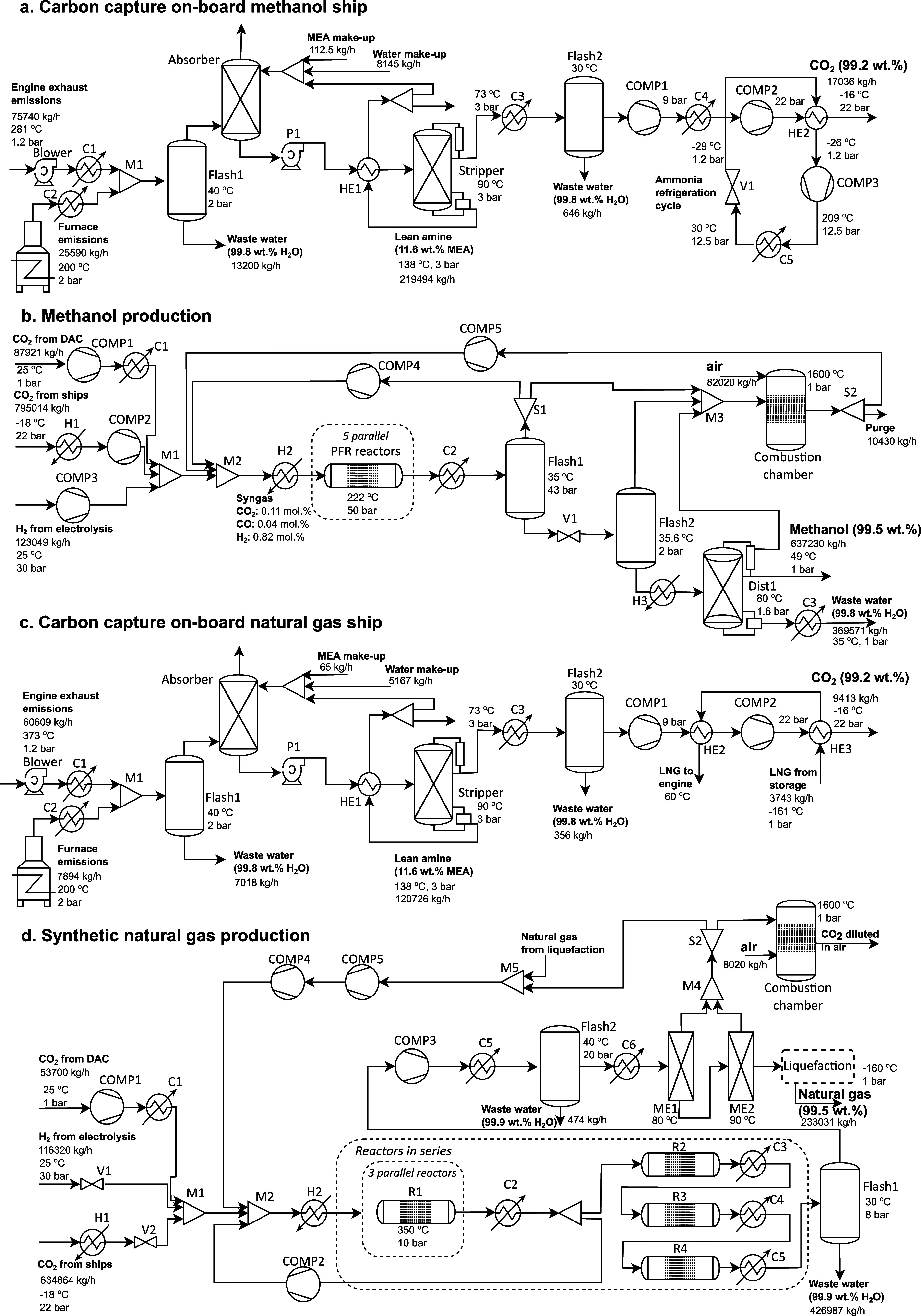
Process flowsheets for circular marine fuels. a. Carbon capture onboard the methanol ship. b. Methanol production. c. Carbon capture onboard the natural has ship. d. Natural gas production.

We develop detailed process models for the two circular marine fuels, dividing the study into two parts; one conducted at sea and the other on land. The first part, carried out during ship operation, focuses on CO_2_ capture and liquefaction. The second part, conducted on land near the port, covers fuel production, separation, and conditioning to meet storage requirements. Both processes are designed based on literature data and tailored to the specific characteristics and limitations of each ship. The process simulations for onboard carbon capture and methanol production were performed using Aspen HYSYS v12.1, while natural gas production was modeled in Aspen PLUS v12.1.^36,37^ All simulations are heat integrated using the Aspen Energy Analyzer.^38^ Detailed information on the process simulations can be found in the Supporting Information (SI). Figure 2 presents the process flowsheets developed for this work.

For the carbon capture part, we do not consider exhaust cleaning because the synthetic propellants are expected to produce negligible sulfur oxides (SO_*x*_), nitrogen oxides (NO_*x*_), and particulate matter (PM) emissions during combustion, as supported by the literature.^4,16^ As a result, these fuels comply with the current emission regulations.^16^ Moreover, the solvent used in the carbon capture process is not at risk of degradation from SO_*x*_, eliminating the need for a dedicated scrubber to neutralize SO_*x*_ emissions.^39^ Our carbon capture plant follows a conventional post-combustion design with a high technology readiness level. This design can be easily retrofitted onboard ships without significant changes or additional space requirements, beyond what is required for fuel switching, as reported by Stolz et al.^40,41^

We assume complete combustion for both methanol and natural gas ships, using specific air-to-fuel ratios and temperatures. The exhaust stream from the methanol engine exits at 281 °C,^42^ containing 18 wt % CO_2_, while the natural gas exhaust stream exits at 373 °C with 15 wt % CO_2_.^43^ The exhaust streams mix with the flue gases from the furnace, which is designed to cover the heat requirements of the system that cannot be covered by the engine waste heat, and then sent to a flash unit that separates the water produced during combustion. The resulting mixed exhaust stream enters the bottom of the absorption column and a 30 wt % aqueous solution of monoethanolamine (MEA) the top to absorb CO_2_, forming a CO_2_-rich solution leaving at the bottom. The remaining CO_2_ is released into the atmosphere from the top of the column.

The CO_2_-rich stream is then pumped and heated to 90 °C before entering a second column, where the CO_2_ is desorbed by heat, resulting in a CO_2_-lean gas leaving at the top, while the MEA solution exits at the bottom and is recycled back to the top of the absorption column. The MEA solution is cooled to 40 °C and mixed with fresh MEA and water to compensate for system losses and maintain the required concentration in the absorber. The heat required for desorption is supplied by medium pressure steam generated in a furnace using the propellant fuel, with a heat consumption rate of 4.9 MJ kgCO_2_^–1^. The CO_2_-lean stream, exiting the desorber at 73 °C, undergoes a series of compression steps with intermittent cooling and a refrigeration cycle to reach the storage conditions of 22 bar and −18 °C.^44^ For the methanol ship, we design a refrigeration cycle using ammonia, while for the natural gas ship, we utilize the refrigeration temperatures already needed for storage by integrating the heat from the CO_2_-lean stream to heat up the natural gas before it is sent to the engine and the furnace, achieving the necessary cooling to reach the desired conditions.

For the methanol production plant, we develop a process simulation based on the work of González-Garay et al. (Figure 2b).^45^ After the captured CO_2_ is unloaded at the port, it is transported to the fuel production facility. There, the CO_2_ is vaporized, compressed to 50 bar, and then mixed with electrolytic H_2_ and additional CO_2_ coming from DAC. The mixed stream reacts under a Cu/ZnO catalyst at 222 °C using 5 reactors in series. The reactor outlet is fed to a series of two flash units to remove the light gases at the top and recycled back to the reactor inlet, while the liquid output consists of a methanol–water solution. This solution is fed into a distillation column at 80 °C and 1.6 bar, where 99.5 wt % methanol is obtained at 49 °C. Light gases from the flash units and distillation column are sent to a combustion chamber where CO_2_ is generated and recycled back to the reactor inlet.

The production of natural gas was designed based on the process simulation developed by Chauvy et al. (Figure 2d).^46^ The process is divided into two parts; the methanation and liquefaction, although here we focus on the methanation process. For the methanation, CO_2_ is mixed with H_2_ and sent to a reaction system comprising of four fixed-bed adiabatic reactors operating in series. Given that methanation is a highly exothermic reaction, multiple reactors in series are necessary to control the temperature. In the first reactor, to keep the outlet temperature below 650 °C, 51% of the outlet stream is recycled back to the inlet, resulting in a very large flow. This high mass flow is then separated into three equal streams, each entering one of the three parallel reactors operating at 10 bar and 350 °C. After the reaction system, the natural gas stream is dehydrated through two flash units. Then, the stream of 86 wt % natural gas is separated from H_2_ and CO_2_ using two membranes operating at 80 and 90 °C. The purified natural gas stream (98.4 wt %) is sent to the liquefaction section, where it is conditioned to −160 °C. The liquefaction process is designed based on the most efficient large-scale natural gas liquefaction process, namely AP-X, developed by Air Products.^47^

### Economic and Technical Feasibility Assessment

Since the purpose of the ships remains the same, i.e., transport of cargo, we ensure that the retrofitted equipment would not affect greatly the operation of the ships. First, we determine the technical feasibility of our approach by assessing the percentage of the ship’s weight and volume taken up by the retrofitted equipment, similar to Negri et al.^28^ We calculate the additional weight relative to the ship’s dead weight tonnage (DWT), assuming that the final weight should remain constant, and estimate the number of TEUs that would need to be displaced to make room for the new equipment.^48^ The weight and volume of the carbon capture plant are estimated based on the design specifications of each equipment, including the absorber, desorber, furnace, flash units, compressors, heat exchangers, and tanks for MEA, ammonia, CO_2_ and the heating fuel. Additional details on the calculation of cargo displacement is provided in the SI. Moreover, we calculate the fuel penalty due to the additional fuel required for heating.

For the economic assessment, we use correlations and installation factors from Towler and Sinott to estimate the capital costs (CAPEX), while for operating costs (OPEX) we consider the costs of raw materials and energy flows.^49^ For the electricity and fresh water required onboard, we assume that it will be provided by a shaft-generator and a desalination system, respectively.^50^ All costs are adjusted to the Chemical Engineering Plant Cost Index of 2023.^51^

### Environmental Assessment

We perform pLCA following the LCA guidelines described in the respective ISO standards.^52,53^ The goal of our LCA is to calculate the future global warming (GW) impacts of methanol and natural gas as circular marine fuels and compare them to the BAU. We consider as functional unit (FU) one tonne kilometer (tkm), which is the basis for assessing the performance of freight transport modes. We adopt a cradle-to-propulsion-to-cradle scope including all upstream activities from the fuel production, to the use of the fuel in the engine of the container ship and furnace and the utilities required for the capture onboard. The system boundaries cover the construction and maintenance of the ship, and port facilities.

The inventories are built in brightway2 v2.4.6 using the graphical user interface activity browser v2.10.2.^54,55^ The foreground system is modeled based on the mass and energy flows taken from the process simulations and literature data for the electrolytic H_2_ and CO_2_ from DAC. Data on electrolytic H_2_ are based on Bareiß et al.,^56^ while data for gasket materials and membrane construction are retrieved from Koj et al. and Evangelisti et al., respectively.^57,58^ For the atmospheric CO_2_, the DAC plant is modeled based on Keith et al.^59^ To develop the prospective life cycle inventories, we use premise v2.1.3.^31^ To model our prospective background system, we use ecoinvent v3.10 and the results of REMIND IAM scenario for the SSP2 (“Middle of the road”) following the RCP2.6 (2 °C warming until 2100).^60−62^ The foreground system is modeled based on the ecoinvent activity named “transport, freight, sea, container ship”, which assumes a similar size ship to the one we considered in our work fueled with HFO (BAU scenario). For methanol and natural gas, we modify the inventory to consider their consumption as well as the emissions after capturing CO_2_. More details on the emissions considered in the environmental assessment and a complete list of activities included in the environmental assessment can be found in the zenodo repository.

For the impact assessment, we use the IPCC 2021 global warming (GW) potentials for a 100-year time horizon expressed in kgCO_2-eq_.,^63^ while the calculations are performed in brightway v2.4.6. For the interpretation phase, we analyze the global warming impacts of our scenarios in 2030 and 2050, evaluating the potential of our developed circular marine fuels to achieve the emission reduction targets set by the maritime industry by 2050.

## Results and Discussion

### Technical and Economic Results

Both circular marine fuel scenarios; carbon capture onboard methanol ship and natural gas ship, are technically feasible. They would increase the overall costs by a factor of 3–6 compared to current operations, with total capital expenses of 85 million USD for methanol and 63 million USD for natural gas. The developed designs expected to be retrofitted onboard ships have a net carbon efficiency of 91%, considering the CO_2_ from the engine exhaust and the furnace. To achieve this, the added weight of the capture unit and the captured CO_2_ would take up to 8% of DWT for the methanol ship and 5% for the natural gas ship — for a week trip, where the stored CO_2_ is 51–60% of the added weight. Additionally, from a volume perspective, methanol will take an equivalent space of 10% of TEU (335 TEU), and natural gas, 6% (206 TEU), with tanks and flash units taking up 97% of that space. Even if the space requirement is considerable, several studies have investigated the topology onboard ships concluding that is feasible to place the added equipment.^20,21^

However, in order for the DWT to remain the same, cargo should be displaced and redistributed to other ships traveling the same route with lower loads without disrupting the existing supply chain or needing new ships, which will further increase the costs. Since our calculations are performed for a week trip, we expect that for longer voyages (of up to 30 days considering the route of East Asia to Europe),^64^ heavier and larger equipment is required rendering the carbon capture onboard technically challenging. A sensitivity analysis of capture rates versus cargo displacement for a range of voyage lengths would be needed to further determine the viability of longer trips, however, this is omitted here as it is beyond the scope of the work.

Capturing CO_2_ using the retrofitted carbon capture unit onboard for the two assessed ships will cost 143–349 USD tonCO_2_^–1^ for methanol and 225–328 USD tonCO_2_^–1^ for natural gas calculated using an annual utilization factor of 0.85, equivalent to the operational time of container ships.^65^ These costs are much higher compared to the carbon capture cost of power plants or from conventional ships powered with HFO,^28,66^ although closer to literature results of works that looked at carbon capture for natural gas ships with costs ranging between 93–231 Euro per tonCO_2_.^21,22^ Moreover, our capture costs fall close to the cost of CO_2_ from DAC (341 USD tonCO_2_^–1^ for high-temperature liquid sorbents), indicating that investing in DAC could also be a viable alternative.

**Figure 3:**
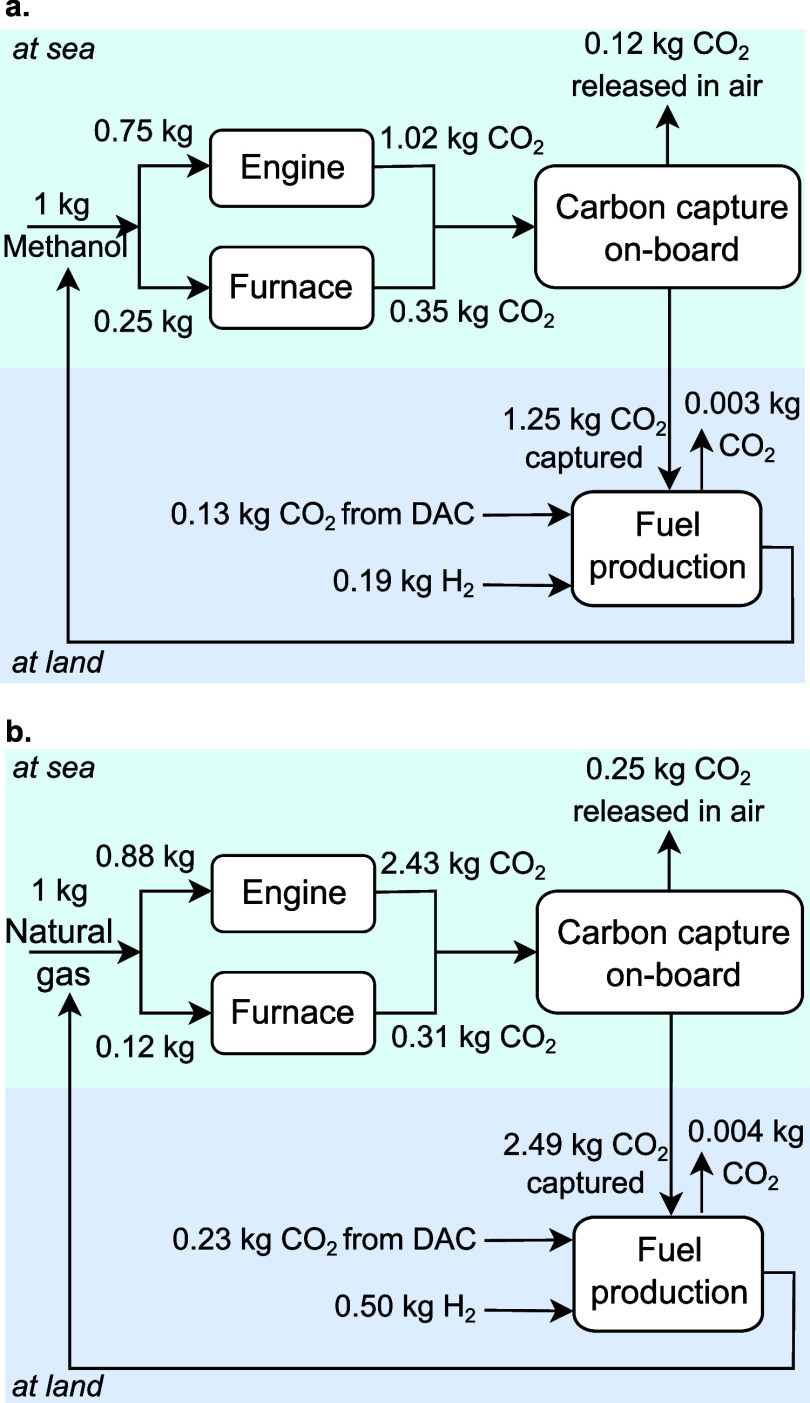
Schemes illustrating the mass balances for the circular carbon fuels. The fuel is used in the engine and the furnace, while the produced carbon dioxide (CO_2_) emissions are combined and sent to the retrofitted carbon capture unit. The captured CO_2_ is stored and after being offloaded at the port is sent to a fuel production facility. Since the CO_2_ is not enough to produce the fuel, direct air capture (DAC) is used to fulfill the stoichiometric amount required. a. Methanol circular fuel. b. Natural gas circular fuel.

Figure 3 illustrates the mass balances of the circular marine fuels concept, displaying that a fuel penalty of 25% is required to cover the heating requirements in the methanol ship (Figure 3a), compared to the 12% penalty in the natural gas ship (Figure 3b). Moreover, the additional requirement for CO_2_ from DAC is 10% for methanol and 8.5% for natural gas, resulting in a cost of 1.12 USD per kg of methanol and 3.4 USD per kg of natural gas. Additionally, even though the H_2_ and CO_2_ requirement per kg of fuel is lower for methanol, its lower gravimetric energy results in 2.7-fold more fuel raising the yearly demands of H_2_ to 984 kton and CO_2_ to 703 kton, as opposed to the 931 ktonH_2_ and 430 ktonCO_2_ for natural gas.

**Figure 4:**
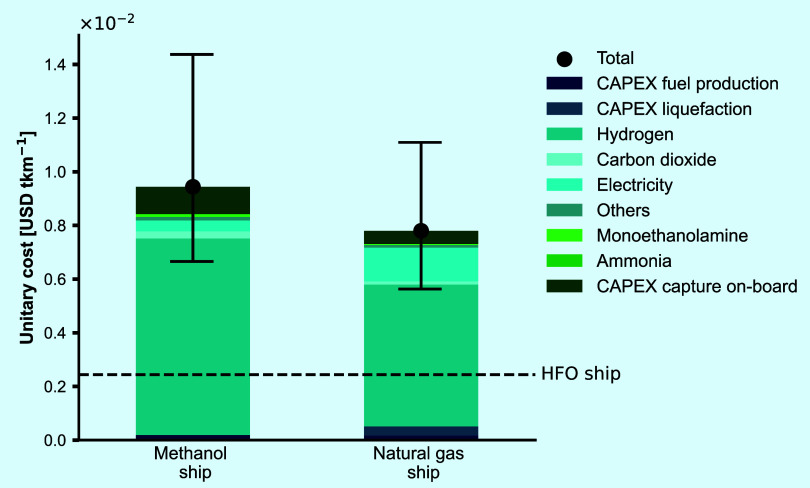
Unitary cost for the two assessed circular marine fuels, expressed in USD tkm^–1^. All values consider the capital (CAPEX) and operating (OPEX) costs for the retrofitted carbon capture plant onboard, for the respective ships, and the fuel production on the land. All values are normalized to USD2023, using the CEPCI. The sensitivity analysis is performed by varying the costs of hydrogen and carbon dioxide from direct air capture. The business-as-usual for the shipping industry (heavy fuel oil — HFO ship) is also plotted for comparison. For the HFO ship we consider the fuel consumption per tkm and the average price of the fuel in Germany for 2023.

Consequently, as shown in Figure 4, natural gas performs better economically with a unitary cost of 7.8 × 10^–3^ USD tkm^–1^ on average compared to the 20% more expensive methanol with 9.4 × 10^–3^ USD tkm^–1^. The costs shown here consider the fuel required for the engine and for steam generation while also the OPEX and CAPEX of the carbon capture process retrofitted onboard the ships, neglecting the cost of building the ships to accommodate the new fuels. Both circular marine fuels show higher costs than the BAU (i.e., 4-fold higher for natural gas and 5-fold higher for methanol) with the cost of H_2_ being the major contributor (78% for methanol and 68% for natural gas). The capture onboard, including CAPEX, and OPEX (heating fuel, MEA, and ammonia), contributes 34% to the methanol ship and 18% to the natural gas ship. Moreover, the CAPEX of carbon capture onboard is 2-fold higher for methanol compared for natural gas due to the higher mass flows that need to be handled. Electricity contributes 16% in the natural gas case with the main consumption required at the liquefaction process, while CO_2_ from DAC contributes only 2.7% in the methanol ship and 1.5% in the natural gas ship. The CO_2_ that is captured onboard is considered free of charge, thus the dependence on the CO_2_ cost from DAC drops significantly.

A sensitivity assessment is also performed to show how much the costs can vary in relation to the fuel price. We used price ranges for Germany, since our fuel production plant is located close to the port of Hamburg. The price range used for hydrogen is between 3–8 USD kg^–1^, and for carbon dioxide between 230–540 USD ton^–1^.^67^ It is evident that even with the lowest prices, circular marine fuels cannot be economically competitive. Using future projections on H_2_ costs of 0.8 USD kg^–1^ until 2050 as projected by Freire Ordóñez et al.,^68^ our circular marine fuels would achieve a cost of 3.4 × 10^–3^ USD tkm^–1^ for methanol and 3.3 × 10^–3^ USD tkm^–1^ for natural gas, for capture costs of 57 USD tonCO_2_^–1^ and 174 USD tonCO_2_^–1^, respectively.

### Environmental Assessment

**Figure 5:**
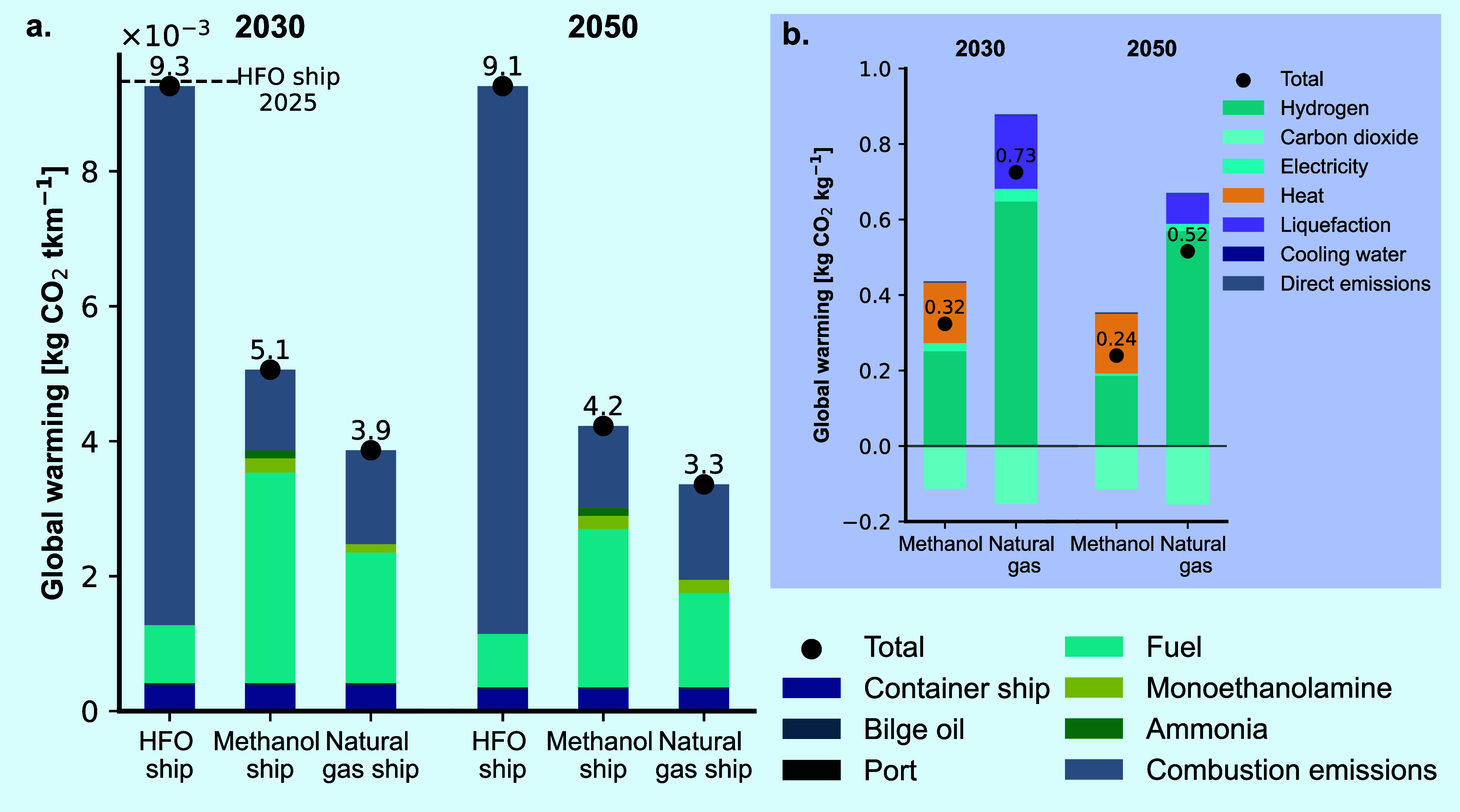
Global warming (GW) breakdown of the studied circular marine fuels and the business-as-usual (heavy fuel oil — HFO ship) in 2030 and 2050 following the 2 °C climate pathway. The system is cradle-to-propulsion-to-cradle since we are recirculating the carbon within the shipping industry. These results consider decarbonization of cement, steel, electricity, and transport respective to the results of REMIND IAM. (a) The GW impacts of the three ships per tonne kilometer (tkm). (b) GW impact of the two circular marine fuels per kg of fuel.

Figure 5a provides the environmental impact breakdown on GW (kg CO_2_ tkm^–1^) for 2030 and 2050, considering the two circular marine fuels (methanol, natural gas), compared to the GW impact of the BAU (HFO ship). The activities shown here consider the use phase (“container ship, “port”, “bilge oil”, “combustion emissions”), fuel production considering the consumption for each specific ship (“Fuel”), and the carbon capture onboard (“monoethanolamine” and “ammonia”), focusing on the operational requirements while assuming that the infrastructure additions related to the carbon capture unit will have minimal impact compared to the respective container ship.

We find that natural gas performs better overall in both years reaching 3.3 × 10^–3^ kg CO_2_ tkm^–1^ in 2050 with a 65% reduction in emissions compared to HFO ship’s 2025 levels, and 58% reduction in 2030. Methanol shows a 55% reduction by 2050 and 45% by 2030. Without onboard carbon capture, emissions from the two alternative powertrains would exceed those of HFO ship. Emission reductions from 2030 to 2050 are mainly due to the integration of renewable technologies across sectors and decarbonization of the electricity mix, both affecting the background data used in the calculations. Onboard capture plays a crucial role in reducing carbon emissions for both short- and long-term maritime goals. Both circular marine fuels can meet the 2030 target of a 20% reduction and approach the 2050 goal of net-zero emissions. Integrating biobased alternative fuels with our developed circular concept could further help the maritime industry meet its’ targets.

Combustion emissions are the largest contributor for the HFO ship, making up 85% of total emissions. The developed approach of fuel switching and onboard carbon capture can tackle 83–85% of these emissions. Despite reductions in combustion emissions, they remain high until 2050. For example, in the methanol ship, combustion emissions account for 23% of impacts in 2030 to 42% in 2050. To further reduce the emissions, we could install a DAC system onboard the ship that will capture the residual emissions, however, this will require additional weight and space on the ship, and higher electricity and heating requirements which will make the implementation extremely challenging. Emissions related to the captured onboard are embedded in ammonia and MEA, contribute 6.5–7.2% for methanol and 5.4–5.7% for natural gas. The container ship’s contribution ranges from 7–12% across scenarios, with a GW impact of 3.2 × 10^–4^ kg CO_2_ tkm^–1^.

Figure 5a shows that fuel production will be the main contributor to GW impacts in both the methanol and natural gas scenarios, compared to only 9.2% for the HFO ship. However, methanol fuel contributes 60% more than natural gas per tkm (looking in the “Fuel” GW impacts), even if per kg of fuel methanol performs 56% better compared to natural gas, as shown in Figure 5b. H_2_ and heating take up 94% of positive GW impacts of methanol, while H_2_ and liquefaction GW emissions contribute 96% of the total positive emissions of natural gas. Decarbonization of electricity decreases the GW impacts of liquefaction by 60% from 2030 to 2050.

CO_2_ from DAC is carbon-negative. However, recall that our system recycles CO_2_ within the shipping industry, so the amount of DAC CO_2_ is reduced relative to the case without utilization of the CO_2_ captured onboard, leading to lower carbon-negative emissions per kg of fuel and resulting in a carbon-positive fuel. Methanol’s carbon-negative potential using only CO_2_ from DAC is −0.61 kgCO_2_ kg^–1^ while for natural gas it is −1.2 kgCO_2_ kg^–1^ (cradle-to-gate emissions). Furthermore, the use of renewable energy to power the DAC unit can further reduce the GHG emissions per ton CO_2_.

### Circularity, CCU, CCS, or Zero-carbon Fuels?

The development of circular marine fuels offers a practical pathway for the maritime industry to meet its short-term 2030 targets while significantly contributing to 2050 goals by capturing emissions from large vessels that will continue operating on carbon-based fuels.^5^ By reusing carbon within a closed-system, this approach can facilitate a smoother transition to more extreme alternatives like zero-carbon options–such as batteries, hydrogen, and ammonia–which require massive infrastructure changes. Considering the IMO’s target, that projects a 5% adoption of alternative fuels by 2030, and the 2023 DNV report highlighting that construction of large-scale facilities for the production of carbon-neutral fuels can take up to a decade, smaller, more easily implemented projects will be prioritized, while onboard carbon capture on these powertrains can be applied as a niche application supporting the goals of the IMO in the short term.^5^

Additionally, carbon capture onboard is being explored as a short-term solution for conventional HFO ships.^18^ This retrofitted infrastructure could be adapted for low-carbon powertrains in the future. Moreover, the reduced reliance on CO_2_ from DAC for fuel production is crucial to the developed concept, as DAC has not yet been implemented on large scale, making its’ future availability unpredictable. Additionally, competition for CO_2_ among hard-to-abate sectors, such as the chemical industry, further limits its availability for the maritime industry. Compared to a CCU alternative based only on CO_2_ from DAC, capture at point sources, i.e., cogeneration plants, cement plants, could result in lower fuel costs and ensure a steady supply of CO_2_, although additional transportation costs of around 20 EUR tonne^–1^ could increase the costs.^69^ We clarify that including other CO_2_ sources in the proximity of the fuel production facilities for potentially reducing costs is out of the scope of this work.

H_2_ remains the primary cost driver and source of life cycle emissions, indicating that e-fuels might not be the definitive solution for shipping. However, by establishing agreements with fuel production companies and hydrogen producers the shipping industry can potentially secure fuels at a lower cost, leveraging their role as a major supplier of CO_2_. The swift licensing and expansion of renewable power plants for scaling up electrolytic H_2_ production are required to support the supply chain of this circular concept, and should be supported by renewable financial investments. In addition, biobased alternatives are appealing due to their biogenic carbon content and simpler logistics,^32^ but their attractiveness spans through various sectors including chemicals,^70^ plastics,^71^ and electricity,^72^ leading to uncertainty in biomass availability and sectoral prioritization. E-fuels offer advantages over biofuels in the transport sector–such as higher engine efficiency, and cleaner combustion emissions. Moreover, biofuels may require additional onboard equipment to control SO_*x*_ and NO_*x*_ emissions, making carbon capture onboard more complex. Without the circular concept, even with CO_2_ capture onboard from e-fuels or biobased fuels, achieving negative cradle-to-grave emissions would necessitate robust CCS value chains.

Methanol and natural gas are currently the most promising alternative fuels for the shipping industry and the primary candidates for the circular carbon concept. However, competition for methanol and natural gas from the chemical industry^73^ and heating sectors^74^ could potentially disrupt the supply of green e-fuels to the shipping industry. Nevertheless, switching to these fuels will be crucial for reaching the 5–10% target for alternative powertrains until 2030.^4^ To effectively integrate these fuel production pathways, a detailed analysis that considers both economic and environmental implications is pivotal.

In conclusion, the transition to a carbon-neutral shipping industry might be gradual, with conventional ships still constituting a big share of the fleet even by 2050. While alternative powertrains of natural gas, methanol, and ammonia are emerging, a diverse portfolio of technologies and fuel production pathways will be essential for a successful transition. Consideration of environmental, economic, and sectoral competitiveness factors is thus critical to find the best portfolios of technologies in the sustainable transition.

## Conclusions

Here, we performed a technoeconomic and environmental assessment of circular marine fuels, incorporating carbon capture onboard ships powered by CO_2_-based fuels. The captured CO_2_ is stored onboard until the ship reaches the next port, where it is unloaded and transported to a fuel production facility where it is converted back into the propulsion fuel. We assessed methanol and natural gas as potential circular marine fuels, and compared them to the BAU scenario. Our findings suggest that this is a viable pathway to meet the emission reduction targets of the maritime sector, although it comes with significant cost increases (3 to 6-fold increase relative to the BAU).

We also find that implementing carbon capture onboard CO_2_-based ships would incur carbon capture costs of 143–349 USD tonCO_2_^–1^ for methanol and 225–328 USD tonCO_2_^–1^ for natural gas. The retrofitted equipment would occupy 8% of the total tonnage capacity for methanol ships and 5% for natural gas ships. Additionally, to desorb the CO_2_ onboard, a fuel penalty would be necessary to cover heating needs–amounting to 25% for methanol and 12% for natural gas. We also found that only 10% of CO_2_ for methanol and 8.5% for natural gas would need to be outsourced from DAC. Lastly, our prospective LCA shows a 65% reduction in GW compared to the BAU for the natural gas ship by 2050 and a 55% reduction for methanol, demonstrating that this approach can enable the sector to meet its emission reduction goals.

Based on our results, circular marine fuels present a flexible and viable option for transitioning to a carbon-neutral shipping industry. This approach ensures the availability of carbon while it can benefit from IMO’s carbon credits and the upcoming legislative changes that will require more expensive emissions allowances for the sector, incentivizing shipowners to adopt decarbonizing technologies.^75−77^ Moreover, it could be implemented with minor modifications beyond those required for fuel switching. Future research should aim to identify a diverse portfolio of technologies and fuel production pathways, taking into consideration environmental impacts, economic viability, and cross-sectoral competitiveness.

## Supporting Information Available

Information on the process parameters, design and cost assumptions, and the weight and volume of the equipment. Also, a zenodo repository has been created for this publication and it can be accessed through this link: 10.5281/zenodo.13900075 (PDF)

## Notes

The repository includes information on the environmental and economic data, as well as the process simulations developed for this work.
