# Supplementary material for: Onboard Carbon Capture for Circular Marine Fuels
Source: ACS Sustain Chem Eng. 2025 Feb 4;13(10):3919–29. doi: 10.1021/acssuschemeng.4c08354 (PMC11921038; doi:10.1021/acssuschemeng.4c08354)
Supplement: Supplementary file 1 — sc4c08354_si_001.pdf [file sc4c08354_si_001.pdf]

Supporting Information

Onboard carbon capture for circular marine fuels

Margarita A. Charalambous,[†] Valentina Negri,[†] Valentin Kamm,[†] Gonzalo Guillén-Gosálbez[†,]*

[†]Institute for Chemical and Bioengineering, Department of Chemistry and Applied Biosciences,

ETH Zurich, Vladimir-Prelog-Weg 1, 8093 Zurich, Switzerland.

[*]Corresponding author: Gonzalo Guillén-Gosálbez: gonzalo.guillen.gosalbez@chem.ethz.ch.

This document includes additional material to the content presented in the main article. Here we report process parameters, design and cost assumptions, and the weight and volume of the equipment.

Number of pages: 33

Number of tables:12

Number of Figures:1

Table of contents

1. Combustion parameters 3

2. Process simulations 4

   2.1. Carbon capture onboard methanol ship 8

   2.2. Methanol production 9

   2.3. Carbon capture onboard natural gas ship 11

   2.4. Natural gas production 12

   2.5. Heat integration 14

3. Economic assessment 15

   3.1. Capital costs 15

   3.2. Operating costs 27

4. Cargo displacement 28

5. References 31

1. Combustion parameters

This section provides more detailed information on modeling the combustion in the engine of the two container ships considered in this work: i) Main parameters used (**Table S1**), combustion equations (**Eq. (1), Eq. (2)**), combustion emissions (**Table S2**).

Table S1. Parameters used for the methanol and natural gas combustion.

Parameter	Methanol	Natural gas	References
Low heating value [MJ/kg]	20	50	1
Engine efficiency [%]	41	50	
Air-to-fuel ratios	6.5	17.3	2,3
Engine size [kW]	23000	23000	4

We assume that the combustion is complete, so the fuel is fully converted to water and carbon dioxide (CO_2). Below the combustion equations are shown, **Eq. (1)** for the methanol engine, and **Eq. (2)** for the natural gas engine.

$$CH_3OH + 1.5\ O_2 \rightarrow CO_2 + 2\ H_2O \quad (1)$$

$$CH_4 + 2\ O_2 \rightarrow CO_2 + 2\ H_2O \quad (2)$$

Following the information from **Table S1** and **Eq. (1)** and **(2)** we model the exhaust gas composition. **Table S2** includes the composition of the exhaust gases from the two engines. Here, we discard the emissions of nitrogen oxides, sulfur oxides, and particulate matter that are important emissions in the case of fossil fuel. E-fuels can yield cleaner combustion with near zero sulfur oxides and particulate matter, while nitrogen oxides are reduced by 80 % compared to emissions from heavy fuel oil.[5] Consequently, our design does not require aftertreatment before sending the flue gases to the capture unit. Additionally, onboard carbon capture can be more easily

implemented on ships that operate with e-fuels, as the amine solvent is not degraded by sulfur oxides, eliminating the need for additional equipment to remove them.

Table S2. Exhaust gas composition from the two container ships

Parameter	Methanol [mole %]	Natural gas [mole %]
CO_2	11.5	9.45
N_2	64.6	70.6
H_2O	23.0	18.9
Argon	0.80	0.89
O_2	0.10	0.10

2. Process simulations

In this section, we include information on the process simulations that were developed for this work. As shown in Figure 2 of the main manuscript we developed four process simulations, two for methanol and two for natural gas. For both fuels we create one simulation for onboard carbon capture and one for fuel production. Here we provide the main assumptions used for the development of each of the process simulations. Moreover, we include: i) The main flows of the developed process simulation for the carbon capture onboard methanol ship (**Section 2.1, Table S4**), ii) methanol production (**Section 2.2, Table S5)** iii) carbon capture onboard natural gas ship (**Section 2.3, Table S6**), iv) natural gas production (**Section 2.4, Table S7**), v) heat integration (**Section 2.5, Table S8**).

The processes have been designed based on the following assumptions:

- The demand for methanol and natural gas are calculated based on the demand of heavy fuel oil in the port of Hamburg, in Germany (1.9 million tonnes based on 2015 data).[6] The amount of heavy fuel oil is then converted to methanol and natural gas using their respective low heating values. The calculated amounts represent the amount of fuels

required for only propulsion used in the engine, 3.8 million tonnes per year for methanol and 1.6 million tonnes for natural gas. The port of Hamburg is the third largest part in Europe making it a good basis for the circular marine concept.

- To calculate the demand for fuel providing heating onboard due to the additional requirements for the reboiler of the desorption column we proceed as follows. We first assume that the maximum amount of heat from the engine exhaust emissions is integrated to cover the reboiler duty, and the rest of the heat is covered using the propulsion fuel. We model combustion in the furnace assuming it operates similarly to combustion in the engine. Using the same equations above (**Eq. (1) and Eq.(2)**) we calculate the exhaust emissions from the furnace. **Table S3** shows the amount of fuel required for each case as well as the exhaust emissions from the furnace.

Table S3. Calculated exhaust composition from furnace, and fuel required for providing heat.

Parameter	Methanol	Natural gas
CO_2 [mole %]	11.5	9.45
N_2 [mole %]	64.6	70.6
H_2O [mole %]	23.0	18.9
Argon [mole %]	0.80	0.89
O_2 [mole %]	0.10	0.10
**Fuel [kg/h]**	**3412**	**431.4**

- The total fuel demand accounts for the fuel required by the engine and furnace, while also accounting for 1 % methane slip in the natural gas ship.
- The ship considered is a 3500 twenty-foot equivalent (TEU) unit container ship. Container ships are considered as the most practical vessels for integrating onboard carbon capture due to their frequent and fixed routes. The 3500 TEU size is a smaller and mid-sized container ship, a size considered crucial for regional and specialized trades. We assume a

similar size ship as the Stena Germanica methanol-powered passenger ferry. This ship is operated on the Gothenburg-Kiel route, and it is the first large passenger ferry to use methanol as a marine fuel. The ship has four Wärtsilä-Sulzer 8ZAL40S engines with a total power of 23000 kW, a design speed of 22 knots, and an exhaust temperature of approximately 280 ^oC. The temperature for the natural gas engine is 373 ^oC. We assume that this passenger ship can operate similarly to the container ship in which we are interested, especially in this case that we are handling the exhaust emissions. The deadweight tonnage of the ship is 51000 tonnes like the SEABOARD VOYAGER ships designed by the German HBHUNTE company.[7]

- The case study of the onboard carbon capture considers a week trip from port to port, which is a typical trip length. A similar length trip (7 days) is traveled by the container ship "CMA CGM GOYA" from Hamburg in Germany to Las Palmas in Spain.[8]
- The CO_2 capture process is performed using a 30% wt. aqueous solution of monoethanolamine, although other CO_2 capture technologies are commercially available. The carbon capture technology we used in this work involves liquid absorbents, and in particular post-combustion using amines. A detailed analysis of all possible alternatives for carbon capture is out of the scope of this work and can be found in several reviews.[9]
- All process simulations are created using ASPEN HYSYS v12.1, except for the natural gas production process, which was modeled in Aspen PLUS v12.1.[10,11]
- There is no exhaust cleaning considered after combustion in the engine since the propellants are synthetically produced, minimizing the production of sulphur oxides, nitrogen oxides, and particulate matter. E-methanol and e-natural gas will comply with current emissions regulations related to these pollutants.[5,12]

- The exhaust gases are compressed to 2 bar before entering the bottom of the absorber. The increased pressure results in higher heat availability during energy integration, helping reduce the energy requirement in the reboiler. Also, at 2 bar the solubility of CO_2 increases in MEA, overall increasing the capture rate. The electricity requirements for the compression are covered by the shaft generator of the engine.[13]
- Methanol is a toxic chemical that can be harmful to marine life, yet at concentrations above 3000 mg/l.[14] Furthermore, methanol is fully miscible in water, allowing it to dilute quickly in case of a spill, thereby reducing the likelihood of high concentration zones that could harm marine life. In case of spills, we assume that appropriate technologies are already in place to mitigate these risks and ensure safety, such as corrosion-inhibiting additives, coatings, and non-metallic materials in pipes and tanks.

2.1. Carbon capture onboard methanol ship

The process simulation for carbon capture onboard the methanol ship is shown in Figure 2a of the main manuscript. Below we show the details of the main flows shown in the Figure (**Table S4**).

Table S4. Temperature, pressures, flows, and composition of the main streams for the process simulation designed for carbon capture onboard methanol ship (Figure 2) of the main manuscript.

Carbon capture onboard methanol ship									
**Parameter**	**Engine exhaust emissions**	**Furnace emissions**	**Waste water 1**	**MEA make-up**	**Water make-up**	**Lean amine**	**Waste water 2**	**Ammonia refrigeration cycle (out COMP3)**	**CO_2**
**Vapour fraction**	1.00	1.00	0.00	0.00	0.00	0.00	0.00	1.00	0.00
**Temperature [ºC]**	281	200	40.0	25.0	25.0	138	40.0	209	− 18.5
**Pressure [bar]**	1.20	2.00	2.00	1.00	1.00	3.10	2.00	14.4	22.0
**Mass Flow [kg h^{-1}]**	757×10^2	256×10^2	132×10^2	113	452	219×10^3	646	515×10^1	171×10^2
**Molar Flow [kgmole h^{-1}]**	274×10^1	925	735	1.84	815×10^1	943×10^1	35.8	735	391
**Molar fractions [mole %]**									
**O_2**	1.10×10^{-3}	1.07×10^{-3}	0.00	0.00	0.00	0.00	0.00	0.00	0.00
**N_2**	0.64	0.64	0.00	0.00	0.00	0.00	0.00	0.00	0.34×10^{-3}
**H_2O**	0.23	0.23	0.99	0.00	1.00	0.88	0.99	0.00	16.0×10^{-3}

**CO_2**	0.12	0.12	0.10×10^{-3}	0.00	0.00	1.02×10^{-2}	0.10×10^{-3}	0.00	0.98
**Argon**	8.00×10^{-3}	8.09×10^{-3}	0.00	0.00	0.00	0.00	0.00	0.00	1.13×10^{-3}
**MEA**	0.00	0.00	0.00	1.00	0.00	0.12	0.00	0.00	0.00
**H_2S**	0.00	0.00	0.00	0.00	0.00	0.00	0.00	0.00	0.00
**NH_3**	0.00	0.00	0.00	0.00	0.00	0.00	0.00	1.00	0.00
**CH_4**	0.00	0.00	0.00	0.00	0.00	0.00	0.00	0.00	0.00

2.2. Methanol production

The process simulation for the methanol production is shown in Figure 2b of the main manuscript. Below we show the details of the main flows shown in the Figure (**Table S5**). The process plant is designed to cover the production of methanol for the engine and the furnace. Both CO_2 from ships is used and from direct air capture, while also we consider that CO_2 from the purges is recycled back to the inlet. The simulation is based on González-Garay et al.,[15] and we model the reactor as an adiabatic fixed-bed multi-tubular reactor with 13939 tubes that have 3.68 cm diameter, 0.5 cm wall thickness, and 0.5 void fraction, and the reactor length is set to 7 m, while the volume is 103.5 m^3. The pressure drop through the reactor is calculated using the Ergun equation.[16,17] The reaction uses a commercial copper and zinc oxide (Cu/ZnO) catalyst and the kinetics are as described in Luyben et al.[16] The catalyst has a solid density of 1775 kg m^{-1}, and the particle diameter was set to 5.5 mm.

Table S5. Temperature, pressures, flows, and composition of the main streams for the process simulation simulations designed for methanol production (Figure 2) of the main manuscript.

**Methanol production**									
**Parameter**	**CO_2 from ships**	**CO_2 from DAC**	**H_2 from electrolysis**	**Syngas**	**Reactor out**	**Air**	**Purge**	**Waste water**	**Methanol**
**Vapour fraction**	0.00	1.00	1.00	1.00	1.00	1.00	1.00	0.00	0.00
**Temperature [°C]**	− 18.5	25.0	25.0	222	271	25.0	25.0	20.0	49.1
**Pressure [bar]**	22.0	1.00	30.0	50.0	43.6	1.00	1.00	1.4	1.05
**Mass Flow [kg h^{-1}]**	795×10^3	879×10^2	123×10^3	692×10^2	692×10^4	219×10^3	104×10^2	369×10^3	637×10^3
**Molar Flow [kgmole h^{-1}]**	182×10^2	202×10^1	610×10^2	804×10^3	764×10^3	943×10^1	368	204×10^2	199×10^2
**Molar fractions [mole %]**									
**CO_2**	0.98	1.00	0.00	0.11	0.09	0.05×10^{-2}	0.21	0.00	$0.5. \times 10^{-2}$
**H_2**	0.00	0.00	1.00	0.82	0.79	0.00	0.00	0.00	0.00
**Methanol**	0.00	0.00	0.00	2.90×10^{-3}	0.03	0.00	0.00	0.01	0.99
**H_2O**	0.02	0.00	0.00	1.00×10^{-3}	0.03	0.00	0.14	0.99	0.00
**CO**	1.13×10^{-3}	0.00	0.00	43.9×10^{-3}	0.05	0.00	0.03×10^{-2}	0.00	0.00
**Argon**	0.00	0.00	0.00	14.3×10^{-3}	0.02	0.13	0.02	0.00	0.00
**N_2**	0.34×10^{-3}	0.00	0.00	3.10×10^{-3}	0.33×10^{-2}	0.76	0.57	0.00	0.00
**O_2**	0.00	0.00	0.00	0.00	0.00	0.23	0.06	0.00	0.00

2.3. Carbon capture onboard natural gas ship

The process simulation for carbon capture onboard the natural gas ship is shown in Figure 2c of the main manuscript. Below we show the details of the main flows shown in the Figure (**Table S6**).

Table S6. Temperature, pressures, flows, and composition of the main streams for the process simulation simulations designed for the carbon capture onboard natural has ship (Figure 2) of the main manuscript.

**Carbon capture onboard natural gas ship**									
**Parameter**	**Engine exhaust emissions**	**Furnace emissions**	**Waste water 1**	**MEA make-up**	**Water make-up**	**Lean amine**	**Waste water 2**	**LNG from storage**	**CO_2**
**Vapour fraction**	1.00	1.00	0.00	0.00	0.00	0.00	0.00	0.00	0.00
**Temperature [ºC]**	373	200	40.0	25.0	25.0	138	30.0	− 162	− 18.6
**Pressure [bar]**	1.20	2.00	2.00	1.00	1.00	3.10	2.70	1.00	22.0
**Mass Flow [kg h^{-1}]**	606 $\times 10^2$	789 $\times 10^1$	702 $\times 10^1$	65.7	517 $\times 10^1$	121 $\times 10^3$	356	374 $\times 10^1$	941 $\times 10^1$
**Molar Flow [kgmole h^{-1}]**	218 $\times 10^1$	285	390	1.08	287	518 $\times 10^1$	197 $\times 10^{-1}$	233	216
**Molar fractions [mole %]**									
**O_2**	1.00 $\times 10^{-3}$	1.07 $\times 10^{-3}$	0.00	0.00	0.00	0.00	0.00	0.00	0.00
**N_2**	0.71	0.64	0.00	0.00	0.00	0.00	0.00	0.00	0.34 $\times 10^{-3}$
**H_2O**	0.19	0.23	0.99	0.00	1.00	0.873	0.99	0.00	0.01
**CO_2**	0.09	0.12	0.10 $\times 10^{-3}$	0.00	0.00	1.02 $\times 10^{-2}$	0.10 $\times 10^{-3}$	0.00	0.98
**Argon**	8.90 $\times 10^{-3}$	8.09 $\times 10^{-3}$	0.00	0.00	0.00	0.00	0.00	0.00	1.48 $\times 10^{-3}$

**MEA**	0.00	0.00	0.00	1.00	0.00	1.17×10^{-1}	0.00	0.00	0.00
**H_2S**	0.00	0.00	0.00	0.00	0.00	0.00	0.00	0.00	0.00
**NH_3**	0.00	0.00	0.00	0.00	0.00	0.00	0.00	0.00	0.00
**CH_4**	0.00	0.00	0.00	0.00	0.00	0.00	0.00	1.00	0.00

2.4. Natural gas production

The process simulation for the natural gas production is shown in Figure 2d of the main manuscript. Below we show the details of the main flows shown in the Figure (**Table S7**). The simulation is based Chauvy et al.,[18] while we model the reactors as adiabatic fixed-bed multi-tubular plug flow reactors in series. We model the reactor with tubes that have 5 cm diameter, and 0.44 void fraction, and the reactor length is set to 3 m. The number of tubes was set such that the gas hourly space velocity is close to 4000 h^{-1}. The first reactors in parallel have 17425 tubes, the second reactor in series has 23558 tubes, the third 22878, and the fourth 22945. The pressure drop through the reactor is calculated using the Ergun equation. The reaction uses commercial Ni/MgAl2O4 (15 wt% Ni) catalyst and the kinetics are as described in Froment et al.[19] The catalyst has a solid density of 2350 kg m^{-1}, and the particle diameter was set to 1.1 cm.

Table S7. Temperature, pressures, flows, and composition of the main streams for the process simulation simulations designed for natural gas production (Figure 2) of the main manuscript.

**Natural gas production**									
**Parameter**	**CO_2 from ships**	**CO_2 from DAC**	**H_2 from electrolysis**	**Waste water 1**	**Waste water 2**	**Air**	**CO_2 diluted in air**	**Natural gas from liquefaction**	**Natural gas**

**Vapour fraction**	0.00	1.00	1.00	0	0	1	1	0	0.00
**Temperature [ºC]**	− 18.5	25.0	25.0	30.0	40.0	25.0	25.0	− 162	− 162
**Pressure [bar]**	22.0	1.00	30.0	7.83	20.2	1.00	1.00	1.00	1.00
**Mass Flow [kg h^{-1}]**	635×10^3	537×10^2	117×10^3	527×10^3	612	922×10^1	973×10^1	980×10^1	235×10^3
**Molar Flow [kgmole h^{-1}]**	136×10^2	125×10^1	582×10^2	293×10^2	33.9	317	368	116×10^1	146×10^2
**Molar fractions [mole %]**									
**CH_4**	0.98	1.00	0.00	3.09×10^{-6}	1.09×10^{-5}	0.00	0.00	0.23	0.99
**H_2**	0.00	0.00	1.00	1.07×10^{-6}	3.92×10^{-6}	0.00	0.00	0.73	0.50×10^{-3}
**CO_2**	0.00	0.00	0.00	5.02×10^{-6}	1.32×10^{-5}	0.51×10^{-3}	0.05	0.02	1.12×10^{-3}
**H_2O**	0.02	0.00	0.00	0.99	0.99	0.00	0.26	0.45×10^{-2}	1.80×10^{-3}
**CO**	1.13×10^{-3}	0.00	0.00	6.79×10^{-11}	2.62×10^{-10}	0.00	0.00	0.16×10^{-3}	3.51×10^{-5}
**Argon**	0.00	0.00	0.00	4.76×10^{-8}	1.58×10^{-7}	0.01	0.01	0.52×10^{-2}	1.49×10^{-3}
**N_2**	0.34×10^{-3}	0.00	0.00	6.54×10^{-3}	2.53×10^{-9}	0.76	0.65	0.17×10^{-2}	0.29×10^{-3}
**Propane**	0.00	0.00	0.00	0.00	0.00	0.00	0.00	0.00	0.00
**Ethane**	0.00	0.00	0.00	0.00	0.00	0.00	0.00	0.00	0.00
**Isobutane**	0.00	0.00	0.00	0.00	0.00	0.00	0.00	0.00	0.00
**n-butane**	0.00	0.00	0.00	0.00	0.00	0.00	0.00	0.00	0.00
**O_2**	0.00	0.00	0.00	0.00	0.00	0.23	0.00	0.00	0.00

2.5. Heat integration

All the process simulations are heat integrated using Aspen Energy Analyzer v12.1.[20] For the carbon capture onboard we assume that the lowest temperature of cooling water is 15 ^{o}C.

Table S8. Heating and cooling utilities after heat integration, and the number for coolers, heaters, and heat exchangers.

**Process simulation**	**Heating duty [kJ h^{-1}]**	**Cooling duty [kJ h^{-1}]**	**No. of Coolers**	**No. of Heaters**	**No. of Heat exchangers**
Carbon capture onboard methanol ship	646×10^{5}	145×10^{-6}	9	2	4
Methanol production	647×10^{7}	481×10^{7}	43	10	80
Carbon capture onboard methanol ship	365×10^{5}	875×10^{5}	8	1	5
Natural gas production	0.00	490×10^{7}	76	0	223

3. Economic assessment

In this section, we provide details on the economic assessment, namely on how to calculate the total annual costs (TAC), including investment and operating expenditures, as well as the unitary cost per tonne kilometer (tkm), which is the unit basis for the freight transport sector, and represents the tonne of goods transported per kilometer. Moreover, we compute the capture costs for the two ships. The costs are calculated based on Towler and Sinnott.[21] The TAC considers the annual capital cost (ACC), and the variable operating costs (VOC). Fixed operating costs related to labor, maintenance, taxes, land, and plant overheads, as well as, the costs of purchasing the new methanol and natural gas ships are omitted. We design a technology that can be deployed by retrofitting the new powertrains to reduce their carbon emissions; therefore, we take into consideration that the ships are already built. In any case, shifting to new powertrains is a requirement by the IMO with the first targets being at 5 % fleet share of renewable fuels until 2030. Therefore, the costs are calculated as shown in Eq.(3).

$$\mathrm{TAC} = \mathrm{ACC} + \mathrm{VOC} \tag{3}$$

The following sections include: i) Detailed analysis on the ACC calculations (**Section 3.1**), ii) Operating costs; data and results (**Section 3.2**), iii) Total costs, and capture costs (**Section 3.3**), iv) Sensitivity analysis (**Section 3.4**).

3.1. Capital costs

The costing of each unit follows the correlations in Towler and Sinnott,[21] while we consider that the retrofitted plant onboard the ships is similar to a standard chemical plant. The purchase costs for each unit are calculated according to **Eq. (4)**.

$$C_e = a + b \times S^n \quad (4)$$

where C_e represents the purchasing cost of the unit, a is a constant factor, b is a proportional size-cost factor, n is the cost exponent, and S is the size factor (characteristic of each unit).

To further estimate the capital costs, we use the factorial method that introduces correction factors for equipment erection, piping, instrumentation and control, electrical, civil, structures and building, and lagging and paint. The equation used to calculate the outside battery limits costs including the installation factors is shown below:

$$C = C_e\,[(1 + f_p)\,f_m + (f_{er} + f_{el} + f_i + f_c + f_s + f_l)] \quad (5)$$

where all installation factors are explained in **Table S9.**

Table S9. Installation factors used in the process simulations designed on land (fuel production), and at sea (capture onboard ships)

		**On land**	**At sea**
**Parameter**	**Explanation**	**Value**	**Value**
f_p	Piping	0.8	0.8
f_m	Material	1/1.3[1]	1/1.3[1]
f_{er}	Equipment Erection	0.3	0.3
f_{el}	Electrical	0.2	0.2
f_i	Instrumentation and Control	0.3	0.3
f_c	Civil	0.3	0
f_s	Structures and buildings	0.2	0
f_l	Lagging and paint	0.1	0.1

Moreover, in addition to the installation factors we consider a location factor for Germany since the fuel production plants are going to be constructed and operated in Germany, and we assume

[1]Materials used for the process units include both carbon steel (f_m=1) and 304 stainless steel. (f_m=1.3).

that the construction of the capture onboard also takes place in Germany. This is necessary since the correlations provided in the book are given for the U.S. Gulf Coast, therefore we need to multiply with the difference in currency exchange rates. The location factor used is 1.11. All the costs are adjusted to USD 2023 using the Chemical Engineering Plant Cost Index (CEPCI), 797.9, compared to the CEPCI of 2010 (used for the cost correlations in Towler and Sinnott)[21] which is 532.9. Lastly, after calculating the cost of the plant (including all the equipment), then the additional investments are also added (offsite costs, contingency changes, and the engineering and construction costs) to get the fixed capital costs. Equation (6) shows the form used to calculate the fixed capital cost.

$$FCC = C \times (1 + f_{off}) \times (1 + f_{ec} + f_{cc}) \tag{6}$$

Where C is the cost of all the equipment (inside battery limits), f_{off} represents the offsite costs (0.3), f_{ec} represents the engineering and construction costs (0.3), and f_{cc} represents the contingency changes (0.1).

We consider also the following assumptions:

- 20 years of lifetime for the fuel production plants and 35 years for the capture onboard (similar to the lifetime of ships). The fuel production plants are operated for 8000 hours per year while the capture onboard for 7446 hours (considering a 0.85 utilization factor for the ships). An interest rate of 0.1 is considered for all the design process plants.
- The costing correlations of some of the flash units are calculated using Turton et al.[22]
- The costing of the membranes required to separate H_2 and CO_2 from methane, is calculated based on the area A. The area A can be calculated from the permeate flow rate of the

component that should be separated, the permeance of the membrane, and the driving force, which is given as the difference in partial pressures of the components of interest. The permeance for the H_2 separation membrane was 100 GPU (Gas permeation unit) and for the CO_2 separation membrane GPU was set to 435. All other parameters required in the costing were extracted from the process simulation.

- All tanks are assumed to be horizontal pressure vessels and their sizing is performed as explained in Towler and Sinnot[21]. For the CO_2 tanks, we consider a maximum volume per tank of 940 m^3 as considered in the study of TMS Tankers Ltd and DNV.[23]

- To calculate the costs per tkm we use a speed of 35 km/h (19 knots), the total annual operating hours, and the total tonnes (51000 tonnes).

- Electricity and cooling water are supplied with the equipment installed already onboard.

- Liquefaction is modeled as the AP-X process, developed by Air Products.[24]

- For all the equipment onboard the ship we consider stainless steel equipment therefore we updated all the costs with the material factor of 1.3.

Table S10. Equipment sizes and capital cost. The capital cost is given for the total time horizon of the lifetime. E-xxx correspond to the heat exchangers estimated in the energy integration.

Equipment label	Equipment type	Value of S	Unit of S	No. of Units	Purschased equipment cost [kUSD 2023]
**A. Carbon capture onboard methanol ship**					
**Furnace**	Cylindrical furnace	18.9	MW	1	496×10^1
**Flash 1**	Vertical pressure vessel	267×10^3	kg	5	284×10^2
**Absorber**	Vertical pressure vessel	173×10^2	kg	1	162×10^1
**-**	Structured PVC packing	154	m^3	1	274×10^1
**Stripper**	Vertical pressure vessel	109×10^2	kg	1	936
**-**	Structured PVC packing	86.7	m^3	1	129×10^1
**Flash 2**	Vertical pressure vessel	984×10^2	kg	1	567×10^1
**COMP1**	Centrifugal compressor	489	kW	1	630×10^1
**COMP2**	Centrifugal compressor	392	kW	1	680×10^1
**COMP3-1**	Centrifugal compressor	300	kW	1	482×10^1
**COMP3-2**	Centrifugal compressor	419	kW	1	537×10^5
**Pump**	Centrifugal	60.6	L/s	1	66.1

**Blower**	Blower	29.2	m3/s	1	21.4
**MEA tank**	Horizontal pressure vessel	349×10^1	kg	1	170
**Methanol tank**	Horizontal pressure vessel	652×10^2	kg	1	159×10^1
**Ammonia tank**	Horizontal pressure vessel	228×10^1	kg	1	131
**CO_2 tanks**	Horizontal pressure vessel	289×10^3	kg	3	166×10^2
**E-100**	Cooler	66.0	m^2	1	176
**E-101**	Heater	50.8	m^2	1	207
**E-102**	Heater	23.9	m^2	1	175
**E-103**	Cooler	24.5	m^2	1	123
**E-104**	Cooler	119×10^1	m^2	1	118×10^2
**E-105**	Cooler	230	m^2	1	315
**E-106**	Cooler	503	m^2	1	494×10^2
**E-107**	Cooler	94.7	m^2	1	164×10^2
**E-108**	Cooler	104	m^2	1	205
**E-109**	Cooler	125	m^2	1	222
**E-110**	Cooler	185	m^2	1	274
**E-111**	HX	879	m^2	1	103×10^1
**E-112**	HX	899	m^2	1	219×10^2
**E-113**	HX	93.6	m^2	1	196
**E-114**	HX	98.0	m^2	1	167
**B. Methanol production**					
**COMP1-1**	Centrifugal compressor	349×10^1	kW	1	259×10^3

**COMP1-2**	Centrifugal compressor	318×10^1	kW	1	346×10^3
**COMP1-3**	Centrifugal compressor	182×10^1	kW	1	355×10^2
**COMP2**	Centrifugal compressor	159×10^2	kW	1	114×10^4
**COMP3**	Centrifugal compressor	295×10^2	kW	1	54.4×10^3
**COMP4**	Centrifugal compressor	353×10^2	kW	2	715×10^3
**COMP5**	Centrifugal compressor	255×10^2	kW	1	496×10^3
**PFR reactor**	Jacketed, agitated	103	m^3	5	370×10^2
**Flash 1**	Vertical process vessel (Turton)	518	m^3	90	208×10^3
**Flash 2**	Vertical pressure vessel	823×10^2	kg	1	199×10^1
**Dist 2**	Vertical pressure vessel	140×10^3	kg	2	869×10^1
**-**	Structured PVC packing	116×10^1	m^3	2	682×10^2
**Combustion chamber**	Cylindrical furnace	0.2	MW	1	786
**E-117**	HX	825	m^2	2	211×10^1
**E-118**	HX	101×10^1	m^2	50	502×10^3
**E-119**	HX	973	m^2	2	704×10^1
**E-120**	HX	35.5	m^2	1	629
**E-121**	HX	128×10^1	m^2	1	774×10^1
**E-122**	HX	126×10^1	m^2	1	916×10^1
**E-123**	HX	104×10^1	m^2	3	258×10^2

**E-124**	Cooler	115×10^{1}.	m^2	2	$219. \times 10^{2}$
**E-125**	Cooler	115×10^{1}	m^2	2	246×10^{2}
**E-126**	Cooler	186	m^2	1	274×10^{1}
**E-127**	Cooler	104×10^{1}	m^2	19	255×10^{3}
**E-128**	Cooler	256	m^2	1	406×10^{1}
**E-129**	Cooler	107×10^{1}	m^2	13	214×10^{3}
**E-130**	HX	100×10^{1}	m^2	8	132×10^{3}
**E-131**	Cooler	230	m^2	1	470×10^{1}
**E-132**	Cooler	230	m^2	1	501×10^{1}
**E-133**	HX	580	m^2	1	115×10^{1}
**E-134**	Cooler	922	m^2	1	194×10^{1}
**E-135**	Cooler	345	m^2	1	805×10^{1}
**E-136**	Cooler	436	m^2	1	104×10^{1}
**E-137**	HX	12.8	m^2	1	295×10^{1}
**E-138**	Heater	465	m^2	5	345×10^{1}
**E-139**	Heater	465	m^2	5	345×10^{1}
**C. Carbon capture onboard natural gas ship**					
**Furnace**	Cylindrical furnace	5.99	MW	1	217×10^{1}
**Flash 1**	Vertical pressure vessel	159×10^{3}	kg	4	147×10^{2}
**Absorber**	Vertical pressure vessel	117×10^{2}	kg	1	985×10^{1}
**-**	Structured PVC packing	100	m^3	1	149×10^{1}
**Stripper**	Vertical pressure vessel	632×10^{1}	kg	1	613×10^{1}

**-**	Structured PVC packing	47.8	m^3	1	710
**Flash 2**	Vertical pressure vessel	472×10^2	kg	1	307×10^1
**COMP1**	Centrifugal compressor	270	kW	1	561×10^1
**COMP2**	Centrifugal compressor	280	kW	1	567×10^1
**Pump**	Centrifugal	33.2	L/s	1	66.1
**Blower**	Blower	27.2	m^3/s	1	28.5
**MEA tank**	Horizontal pressure vessel	209×10^1	kg	1	124
**LNG tank**	Horizontal pressure vessel	289×10^2	kg	1	817
**CO_2 tanks**	Horizontal pressure vessel	239×10^3	kg	2	634
**E-100**	Heater	19.7	m^2	1	169
**E-101**	Cooler	691	m^2	1	671
**E-102**	Cooler	35.8	m^2	1	129
**E-103**	Cooler	374	m^2	1	456
**E-104**	Cooler	32.3	m^2	1	127
**E-105**	Cooler	88.5	m^2	1	160
**E-106**	Cooler	103	m^2	1	204
**E-107**	Cooler	164	m^2	1	255
**E-108**	Cooler	36.6	m^2	1	155
**E-109**	HX	99.6	m^2	1	201
**E-110**	HX	989	m^2	1	233
**E-111**	HX	58.6	m^2	1	171

**E-112**	HX	88.6	m^2	1	161
**E-113**	HX	524	m^2	1	513
**D. Natural gas production**					
**Reactors in parallel (R1)**	Jacketed, agitated	126	m^3	3	221×10^2
**R2**	Jacketed, agitated	170	m^3	1	108×10^2
**R3**	Jacketed, agitated	165	m^3	1	106×10^2
**R4**	Jacketed, agitated	166	m^3	1	106×10^2
**COMP1-1**	Centrifugal compressor	118×10^2	kW	1	325×10^2
**COMP1-2**	Centrifugal compressor	264×10^2	kW	1	509×10^2
**COMP2**	Centrifugal compressor	118×10^2	kW	1	325×10^2
**COMP3**	Centrifugal compressor	264×10^2	kW	1	509×10^2
**COMP4**	Centrifugal compressor	290×10^2	kW	1	537
**COMP5**	Centrifugal compressor	423×10^2	kW	1	666×10^2
**Flash 1**	Vertical pressure vessel	326×10^3	kg	9	795×10^2
**Flash 2**	Vertical pressure vessel	307×10^3	kg	10	841×10^2
**ME1**	Membrane (H_2 separation)	133×10^3	m^2	1	309×10^2
**ME1**	Membrane (CO_2 separation)	231×10^2	m^2	1	438×10^1
**E-112**	Cooler	103×10^1	m^2	13	173×10^2
**E-114**	Cooler	159	m^2	1	274

**E-120**	Cooler	112×10^{1}	m^2	3	438×10^{1}
**E-121**	Cooler	982	m^2	9	114×10^{2}
**E-122**	Cooler	969	m^2	6	750×10^{1}
**E-125**	Cooler	100×10^{1}	m^2	11	142×10^{2}
**E-127**	Cooler	829	m^2	2	212×10^{1}
**E-134**	Cooler	972	m^2	16	201×10^{2}
**E-110**	HX	996	m^2	20	257×10^{2}
**E-111**	HX	825	m^2	2	211×10^{1}
**E-116**	HX	987	m^2	35	446×10^{2}
**E-118**	HX	825	m^2	2	211×10^{1}
**E-124**	HX	104×10^{1}	m^2	2	268×10^{1}
**E-131**	HX	880	m^2	4	452×10^{1}
**E-132**	HX	120	m^2	1	239
**E-133**	HX	661	m^2	1	844
**D. Liquefaction of natural gas**					
**Propane Flash 1**	Vertical Pressure Vessel	258×10^{3}	kg	9	463×10^{2}
**Propane Flash 2**	Vertical Pressure Vessel	237×10^{3}	kg	5	240×10^{2}
**Propane Flash 3**	Vertical Pressure Vessel	268×10^{2}	kg	13	691×10^{2}
**MR Flash 1**	Vertical Pressure Vessel	268×10^{3}	kg	52	276×10^{3}
**MR Flash 2**	Process vessel	587	m^3	27	624×10^{2}
**MR Flash 3**	Vertical Pressure Vessel	234×10^{3}	kg	1	474×10^{1}

**Propane COMP1**	Centrifugal compressor	264×10^2	kW	1	509×10^2
**Propane COMP2**	Centrifugal compressor	260×10^2	kW	2	101×10^3
**Propane COMP3**	Centrifugal compressor	267×10^2	kW	2	102×10^3
**Propane COMP4**	Centrifugal compressor	273×10^2	kW	3	156×10^3
**MR COMP 1**	Centrifugal compressor	272×10^2	kW	5	259×10^3
**MR COMP 2**	Centrifugal compressor	243×10^2	kW	5	243×10^3
**MR COMP 3**	Centrifugal compressor	175×10^2	kW	2	809×10^2
**MR COMP 4**	Centrifugal compressor	284×10^2	kW	2	106×10^3
**MR COMP 5**	Centrifugal compressor	151×10^2	kW	2	747×10^2
**COMP1 (before liquefaction**	Centrifugal compressor	283×10^2	kW	1	530×10^2
**Nitrogen expander**	Expander	139×10^1	kW	13	128×10^1
**Pump**	Pump	114	L/s	1	466
**E-113**	Cooler	124×10^1	m^2	1	163×10^1
**E-115**	Cooler	989	m^2	3	383×10^1
**E-117**	Cooler	963	m^2	3	373×10^1
**E-119**	Cooler	991	m^2	9	115×10^2
**E-123**	Cooler	103×10^1	m^2	5	663×10^1
**E-126**	Cooler	882	m^2	3	340×10^1
**E-128**	Cooler	465	m^2	1	605

**E-129**	HX	552	m^2	1	710
**E-130**	HX	781	m^2	2	200×10^1
**E-131**	Multistream HX	100	m^2	15	274×10^1
**E-132**	Multistream HX	100	m^2	15	274×10^1
**E-133**	Multistream HX	100	m^2	15	274×10^1
**E-134**	Multistream HX	100	m^2	15	274×10^1
**E-135**	Multistream HX	100	m^2	60	110×10^2
**E-136**	Multistream HX	101	m^2	30	551×10^1
**E-137**	Multistream HX	105	m^2	6	115×10^1

3.2. Operating costs

Table S11. Cost parameters used in operating cost calculations. Values are expressed in USD 2023.

**Material/energy flow**	**Unit**	**Avg. value**	**Min value**	**High value**	**Reference**
Material flows					
Hydrogen	USD kg^{-1}	5.00	3.00	8.00	[25]
Carbon dioxide (DAC)	USD kg^{-1}	0.25	0.13	0.5	[26]
Catalyst (methanol)	USD kg^{-1}	108	-	-	[27]
Catalyst (natural gas)	USD kg^{-1}	102	-	-	[18]
Propane	USD kg^{-1}	0.37	-	-	[28]
Light hydrocarbons	USD kg^{-1}	0.52	-	-	[29–31]

Nitrogen	USD kg^{-1}	0.12	-	-	[32]
Monoethanolamine	USD kg^{-1}	1.60	-	-	[33]
Ammonia	USD kg^{-1}	0.23	-	-	[34]
Utilities					
Electricity	USD kWh^{-1}	0.31	-	-	[35]
Cooling water	USD kJ^{-1}	3.78×10^{-7}	-	-	[22]
LP steam	USD kJ^{-1}	2.78×10^{-6}	-	-	[22]

4. Cargo displacement

The weight and volume taken up by the retrofitted equipment is calculated and compared to the ship's DWT and TEU. We assume that the final weight should remain constant, and estimate the number of TEUs that should be displaced to make room for the new equipment. **Figure S1** shows the weight of the equipment required for the captured onboard for the methanol and natural gas ships. Moreover, the weight of methanol for the engine and heating consumed are also included, as well as the weight of CO_2 produced. The calculation of the weight follows **Eq.(7)**.

$$\text{Weight} = \text{Equipment} + \text{Ammonia} + CO_2 - \text{Methanol}_{eng+heat} \quad (7)$$

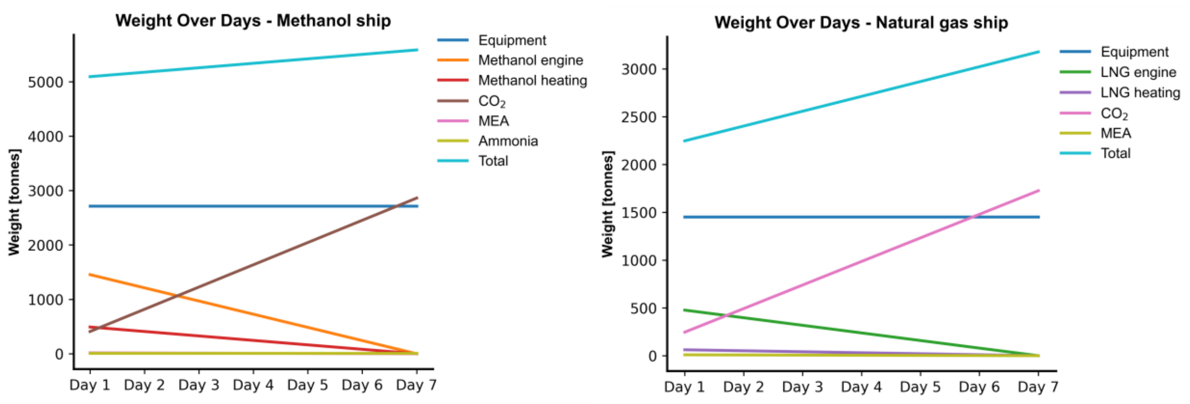

Figure S1. Weight of equipment, fuel for engine and heating, MEA, ammonia, and CO_2 from day1 of the trip to day 7.

Table S12. Weight and volume of all the equipment considered for the capture onboard with methanol and natural gas.

Table S12. Weight and volume of the retrofitted equipment.

**Retrofitted equipment**	**Weight (tonnes)**	**Volume (m^3)**
Capture onboard methanol ship		
Absorber	17.4	156
Desorber	10.9	88.1
Furnace	20	75
Separators	1433	713×10^1
CO_2 tank	866	288×10^1
MEA tank	3.5	19.1
Methanol tank	65.2	754
NH_3 tank	2.28	13.1
Compressors	25.4	61

Capture onboard natural gas ship		
Absorber	11.7	102
Desorber	6.32	48.6
Furnace	20	75
Separators	685	492×10^{1}
CO_2 tank	478	153×10^{1}
MEA tank	2.09	10.9
LNG tank	28.9	162
Compressors	12.7	30.6

We consider the weight of the ship of 8500 TEU, whose deadweight (DWT) is 51000 tonnes. In our calculations, we assume that 1 TEU corresponds to 33 m^3 on average.

5. References

(1) Elis, J.; Tanneberger, K. *Study on the Use of Ethyl and Methyl Alcohol as Alternative Fuels in Shipping*; SSPA SWEDEN AB. https://emsa.europa.eu/air-pollution/alternative-fuels/items.html?cid=329&id=2726 (accessed 2024-09-24).

(2) *The Methanol-Fuelled MAN B&W LGIM Engine*; MAN Energy Solutions. https://www.google.com/search?q=using-methanol-fuel-in-the-man-b-w-me-lgi-series&rlz=1C1GCEA_enCH930CH930&oq=using-methanol-fuel-in-the-man-b-w-me-lgi-series&gs_lcrp=EgZjaHJvbWUyBggAEEUYOTIKCAEQABiABBiiBDIKCAIQABiABBiiBNIBBzc0NWowajSoAgCwAgE&sourceid=chrome&ie=UTF-8 (accessed 2024-09-24).

(3) Kalam, M. A.; Masjuki, H. H.; Maleque, M. A.; Amalina, M. A.; Abdesselam, H.; Mahlia, T. M. I.; Kalam, M. A.; Masjuki, H. H.; Maleque, M. A.; Amalina, M. A.; Abdesselam, H.; Mahlia, T. M. I. Air -Fuel Ratio Calculation for a Natural Gas Fuelled Spark Ignition Engine; SAE International, 2004. https://doi.org/10.4271/2004-01-0640.

(4) *Stena Germanica - Vessel details*. https://stenaline.com/about-us/our-ships/stena-germanica/ (accessed 2024-09-24).

(5) DNV. *Alternative Fuels for Containerships: LNG, Methanol and Ammonia*; 2023. https://www.dnv.com/publications/alternative-fuels-for-containerships-lng-methanol-and-ammonia-242250/ (accessed 2024-08-12).

(6) Union of European Petroleum Independents. Bunkering Facts and Figures. chrome-extension://oemmndcbldboiebfnladdacbdfmadadm/https://www.upei.org/component/flexicontent/download/1033/763/17?method=view (accessed 2024-08-12).

(7) *Sanfu Shipbuilding Delivers Two 3,500 TEU Containerships*. iMarine. https://www.imarinenews.com/13277.html (accessed 2024-09-30).

(8) *CMA CGM | Search port to port Schedules*. https://www.cma-cgm.com/ebusiness/schedules/routing-finder (accessed 2024-09-30).

(9) Muhammad M, F. H.; Manali S., Z.; Monzure-Khoda, K. Challenges and Opportunities in Carbon Capture, Utilization and Storage: A Process Systems Engineering Perspective. **2022**, *166*. https://doi.org/10.1016/j.compchemeng.2022.107925.

(10) *Aspen HYSYS - The Industry's #1 Process Simulation Software*. AspenTech. https://www.aspentech.com/en/products/engineering/aspen-hysys (accessed 2024-08-12).

(11) *Aspen PLUS - The Leading Process Simulation Software in the Chemical Industry*. AspenTech. https://www.aspentech.com/en/products/engineering/aspen-plus (accessed 2024-08-12).

(12) Tripathi, S.; Gorbatenko, I.; Garcia, A.; Sarathy, M.; Tripathi, S.; Gorbatenko, I.; Garcia, A.; Sarathy, M. Sustainability of Future Shipping Fuels: Well-to-Wake Environmental and Techno-Economic Analysis of Ammonia and Methanol; SAE International, 2023. https://doi.org/10.4271/2023-24-0093.

(13) François, M. H. J.-J.; Patil, D.; Brulé, S.; Knuutila, H. K. CO2 Solubility and Amine Volatility Data for Low-Concentration Solutions of MEA, AMP, PZ and CESAR-1 Blend (AMP/PZ). *Results Eng.* **2024**, *22*, 102163. https://doi.org/10.1016/j.rineng.2024.102163.

(14) Methanol Institute. *MARINE METHANOL: Future-Proof Shipping*; 2023. https://www.google.com/search?q=the+MARINE+METHANOL%3A+Future-Proof+Shipping+Fuel+report+by+the+Methanol+Institute+(2023)&rlz=1C1GCEA_enCH930CH930&oq=the+MARINE+METHANOL%3A+Future-Proof+Shipping+Fuel+report+by+the+Methanol+Institute+(2023)&gs_lcrp=EgZjaHJvbWU

yBggAEEUYOdIBCDExNDBqMGo3qAIIsAIB&sourceid=chrome&ie=UTF-8 (accessed 2024-11-06).
(15) González-Garay, A.; Frei, M. S.; Al-Qahtani, A.; Mondelli, C.; Guillén-Gosálbez, G.; Pérez-Ramírez, J. Plant-to-Planet Analysis of CO_2-Based Methanol Processes. *Energy Environ. Sci.* **2019**, *12* (12), 3425–3436. https://doi.org/10.1039/c9ee01673b.
(16) Luyben, W. L. Design and Control of a Methanol Reactor/Column Process. *Ind. Eng. Chem. Res.* **2010**, *49* (13), 6150–6163. https://doi.org/10.1021/ie100323d.
(17) Van-Dal, É. S.; Bouallou, C. Design and Simulation of a Methanol Production Plant from CO2 Hydrogenation. *J. Clean. Prod.* **2013**, *57*, 38–45. https://doi.org/10.1016/j.jclepro.2013.06.008.
(18) Chauvy, R.; Dubois, L.; Lybaert, P.; Thomas, D.; De Weireld, G. Production of Synthetic Natural Gas from Industrial Carbon Dioxide. *Appl. Energy* **2020**, *260*, 114249. https://doi.org/10.1016/J.APENERGY.2019.114249.
(19) Xu, J.; Froment, G. F. Methane Steam Reforming, Methanation and Water-gas Shift: I. Intrinsic Kinetics. *Wiley Online Libr.* **1989**. https://doi.org/10.1002/aic.690350109.
(20) *Aspen Energy Analyzer*. AspenTech. https://www.aspentech.com/en/products/engineering/aspen-energy-analyzer (accessed 2024-08-12).
(21) Towler, G.; Sinnott, R. K. *Chemical Engineering Design - Principles, Practice and Economics of Plant and Process Design*, 2nd ed.; Elsevier, 2013.
(22) Turton, R.; Bailie, R. C.; Whiting, W. B.; Shaeiwitz, J. A.; Bhattacharyya, D. *Analysis, Synthesis and Design of Chemical Processes*; Prentice Hall, 2012.
(23) TMS Tankers Ltd; DNV. On-Board Carbon Capture and Storage for Suezmax Tankers, 2024. https://www.dnv.com/expert-story/maritime-impact/on-board-carbon-capture-and-storage-equipment-feasibility-study/ (accessed 2024-10-02).
(24) Sun, H.; He Ding, D.; He, M.; Shoujun Sun, S. Simulation and Optimisation of AP-X Process in a Large-Scale LNG Plant. *J. Nat. Gas Sci. Eng.* **2016**, *32*, 380–389. https://doi.org/10.1016/j.jngse.2016.04.039.
(25) IEA. Levelised Cost of Hydrogen via Selected Technologies in Northwest Europe in the Announced Pledges Scenario, 2023-2030, 2024. https://www.iea.org/data-and-statistics/charts/levelised-cost-of-hydrogen-via-selected-technologies-in-northwest-europe-in-the-announced-pledges-scenario-2023-2030 (accessed 2024-10-04).
(26) Sievert, K.; Schmidt, T. S.; Steffen, B. Considering Technology Characteristics to Project Future Costs of Direct Air Capture. *Joule* **2024**, *8* (4), 979–999. https://doi.org/10.1016/j.joule.2024.02.005.
(27) Nieminen, H.; Laari, A.; Koiranen, T. CO2 Hydrogenation to Methanol by a Liquid-Phase Process with Alcoholic Solvents: A Techno-Economic Analysis. *Processes* **2019**, *7* (7), 405. https://doi.org/10.3390/pr7070405.
(28) *Propane price in Germany*. LPG price monitoring agency. https://lpg-price.com/propane/germany.html (accessed 2024-10-04).
(29) *Commercial and industrial gas prices Germany 2023*. Statista. https://www.statista.com/statistics/1346785/gas-prices-development-commercial-industrial-customers-germany/ (accessed 2024-10-04).
(30) Alerts, I. *Ethane Prices | Current and Forecast*. Intratec.us. https://www.intratec.us/chemical-markets/ethane-price (accessed 2024-10-04).

(31) Alerts, I. *Isobutane Prices | Current and Forecast*. Intratec.us. https://www.intratec.us/chemical-markets/isobutane-price (accessed 2024-10-04).
(32) *Nitrogen Price in Germany - 2023 - Charts and Tables - IndexBox*. https://www.indexbox.io/search/nitrogen-price-germany/ (accessed 2024-10-04).
(33) Mike. *Monoethanolamine price index*. businessanalytiq. https://businessanalytiq.com/procurementanalytics/index/monoethanolamine-price-index/ (accessed 2024-10-04).
(34) *Ammonia Price in Germany - 2023 - Charts and Tables - IndexBox.* https://www.indexbox.io/search/ammonia-price-germany/ (accessed 2024-10-04).
(35) *Electricity price statistics*. Eurostat. https://ec.europa.eu/eurostat/statistics-explained/index.php?title=Electricity_price_statistics#Electricity_prices_for_non-household_consumers (accessed 2024-10-04).
